# A case of bilateral endogenous endophthalmitis misdiagnosed as Purtscher’s retinopathy

**DOI:** 10.1186/s12886-020-01399-9

**Published:** 2020-04-06

**Authors:** Yanming Huang, Rongdi Yuan

**Affiliations:** Xinqiao Hospital, Army Medical University, 183rd Xinqiao Street, Shapingba District, Chongqing, 400038 China

**Keywords:** Purtscher’s retinopathy, Endogenous endophthalmitis, Cotton-wool spots, Thoracoabdominal trauma

## Abstract

**Background:**

Purtscher’s retinopathy characterized by the appearance of cotton-wool spots and intraretinal hemorrhage at the posterior pole that commonly occurs after severe head and chest trauma. We report a patient who presented with multiple white retinal patches and retinal hemorrhage forty-two days after a severe thoracoabdominal trauma, which was misdiagnosed as Purtscher’s retinopathy.

**Case presentation:**

A middle-aged woman presented to the eye clinic complaining of decreased vision and distortion in the right eye forty-two days after thoracoabdominal trauma. Upon first glance at her fundal appearances with multiple white retinal patches and retinal hemorrhage, we considered it to be bilateral Purtscher’s retinopathy. No specific treatment was given to her. Ten days later, the four white retinal patches in the right eye joined together with star-shaped hard exudates and radial folds in the macula. This was not consistent with the characteristics of Purtscher’s retinopathy. In retrospect, we found that the onset time, shape, and location of the white retinal patches were not cotton-wool spots. A detailed history revealed that she had *Staphylococcus aureus* septicaemia due to abdominal incision infection, and she underwent intravenous antibiotic therapy. Fundus fluorescein angiography (FFA) revealed hyperpermeable vasculature and extensive fluorescence leakage in the middle and late stages. Optical coherence tomography (OCT) revealed highly reflective exudates in the neuroepithelium and macular edema in the right eye. Taking her history and the FFA and OCT results into consideration, she was diagnosed with bilateral endogenous endophthalmitis.

**Conclusion:**

In the present case, multiple white patches and intraretinal hemorrhage at the posterior pole forty-two days after the trauma were not Purtscher’s retinopathy. It was bilateral endogenous endophthalmitis. The subretinal abcesses that developed secondary to *Staphylococcus aureus* infection involved the macula causing decreased vision and distortion in the right eye. We concluded that in the case of multiple white retinal patches at the posterior pole in patients after trauma, especially in patients with infectious disease, Purtscher’s retinopathy is not the only possible diagnosis. Correct diagnosis depends on reevaluation of the lesions by FFA and OCT, laboratory investigation and detailed history.

## Background

Purtscher’s retinopathy is a hemorrhagic and vasoocclusive retinopathy that was first described in 1912 as a syndrome of sudden blindness associated with severe head trauma [1]. Later, it was observed with other types of traumatic injuries, including crush injuries, chest trauma, seatbelt and airbag injuries [2]. It is characterized by cotton-wool spots and retinal hemorrhages around the optic nerve head and fovea [2]. The pathology of Purtscher’s retinopathy is not fully understood. Occlusion of peripapillary terminal arterioles by various embolic particles might be the cause of the striking fundal abnormalities in Purtscher’s retinopathy [3]. It is always associated with decreased visual acuity, central and paracentral visual defects and annular scotoma [3]. FFA features of Purtscher’s retinopathy include capillary nonperfusion, focal areas of arteriolar occlusion, paravascular staining and leakage from the optic nerve head. OCT reveals inner retinal hyperreflectivity at the site of cotton-wool spots [4]. After four to 6 weeks, the cotton-wool spots fade away, and eventually, the fundus may appear normal. We herein report a case of bilateral endogenous endophthalmitis misdiagnosed as purtscher’s retinopathy.

## Case presentation

**Fig. 1:**
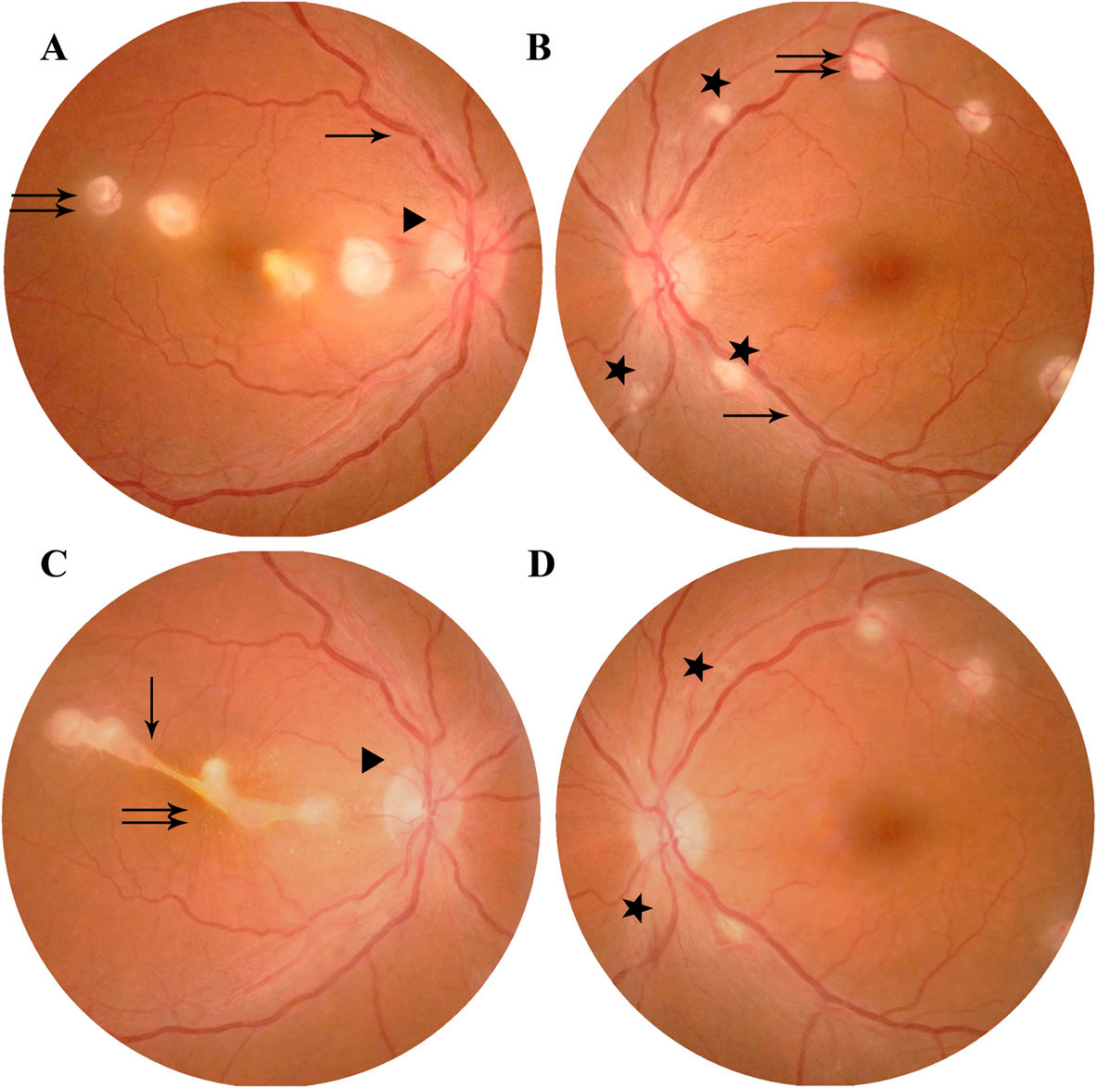
Fundus photographs of both eyes on presentation (**a** and **b**) and ten days later (**c** and **d**) in a woman who complained of decreased vision and distortion in the right eye forty-two days after thoracoabdominal trauma. On presentation, dilated veins (**a** and **b**, arrow) and oval white retinal lesions (**a** and **b**, double arrows) were observed in both eyes. In addition, linearly shaped hemorrhage (**a**, triangle) and cotton-wool spots (**b**, star) were found at the posterior pole. Ten days later, the four separated oval retinal lesions joined together (**c**, arrow) with star-shaped hard exudates and radial folds (**c**, double arrows) in the macula of the right eye. At the same time, the linear hemorrhage (**c**, triangle) disappeared. In the left eye, the two cotton-wool spots near the optic disc faded away (**d**, star)

**Fig. 2:**
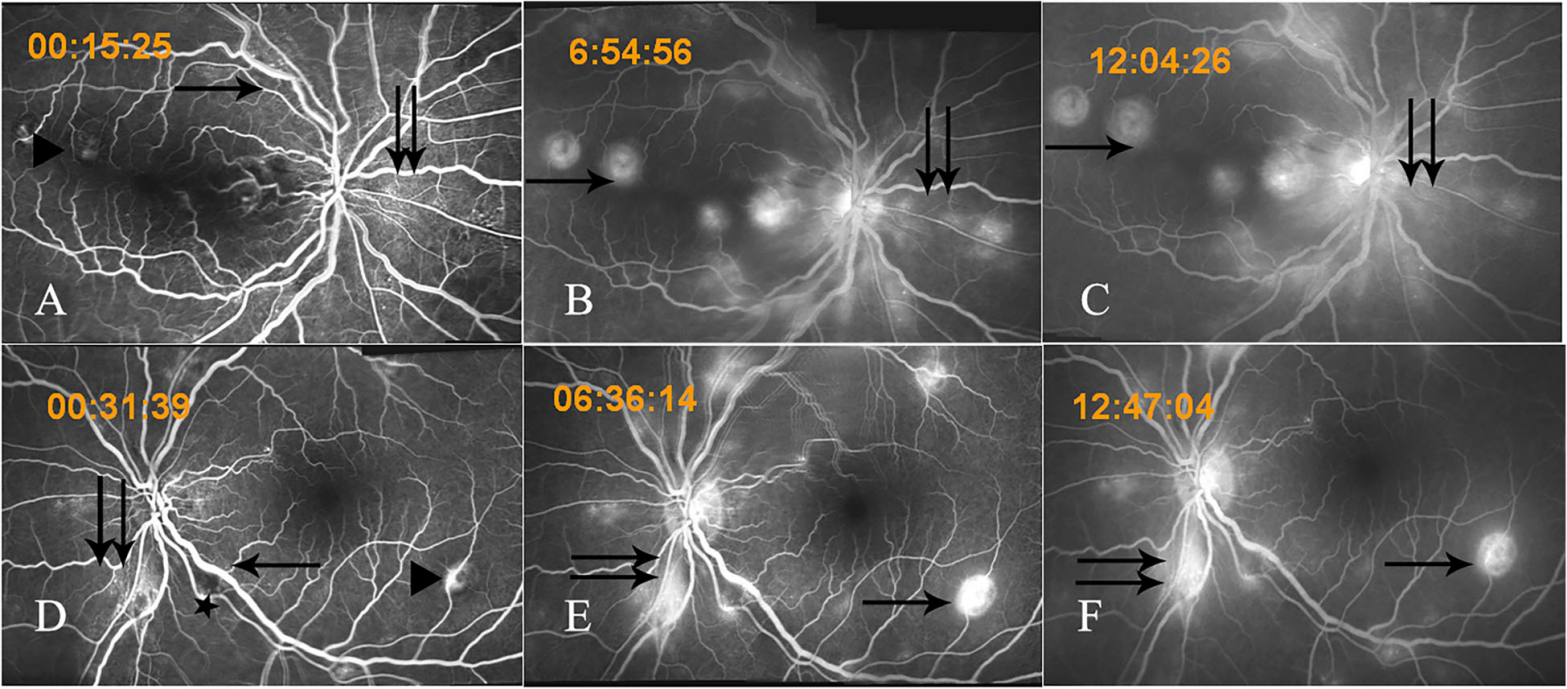
Fundus fluorescein angiography (FFA) of both eyes on presentation. FFA showed dilated retinal veins and capillaries (arrow), less fluorescence leakage at the posterior pole (double arrows) and at the site of the oval retinal patches (triangle) at the early stage (**a** and **d**). At the same time, blocked fluorescence by linear hemorrhage was found in the right eye (**a**). A nonperfusion area (star) at the posterior pole was found in the left eye (**d**). At the middle and late stages of FFA, extensive fluorescence leakage around the optic disc (double arrows) and the site of oval retinal patches (arrow) were observed in both eyes (**b**, **c**, **e** and **f**)

**Fig. 3:**
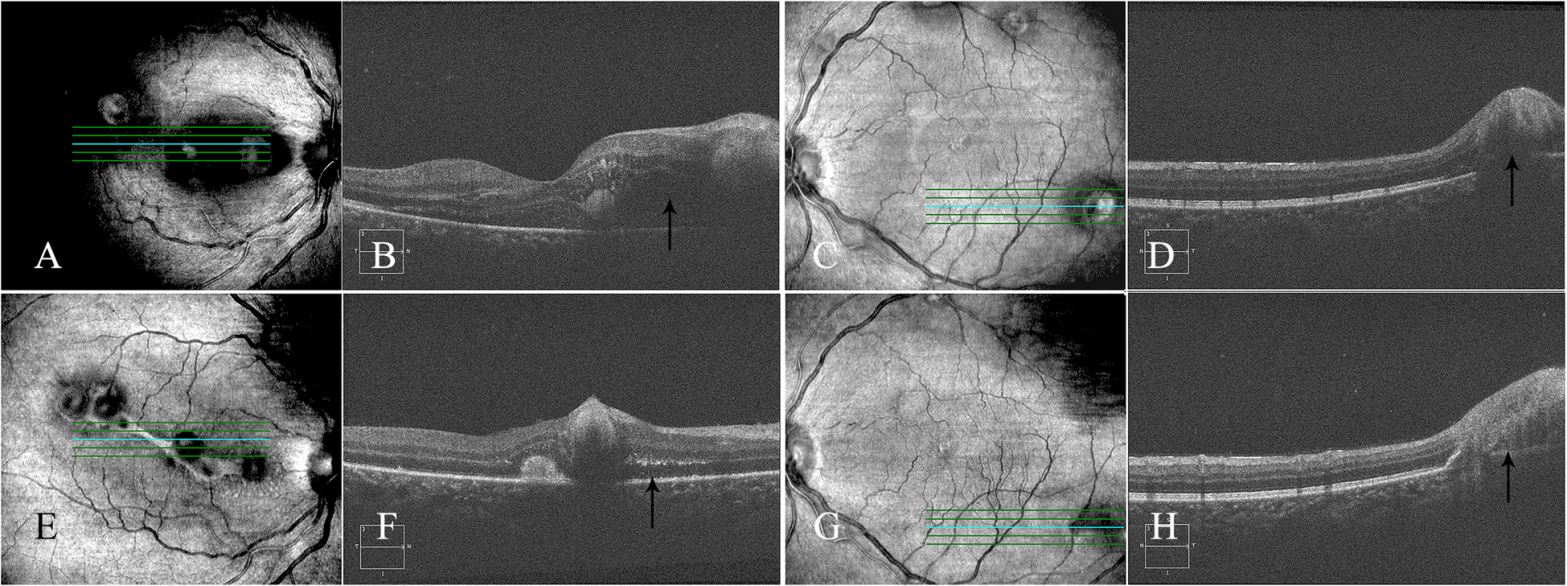
Optical coherence tomography (OCT) of both eyes on presentation (**a, b, c** and **d**) and ten days later (**e, f, g** and **h**). On presentation, macular edema with significant thickening (arrow) nasal to the fovea in the right eye was revealed (**b**). The fovea of the left eye was not involved. At the site of oval retinal patches, the sensory retina was elevated (arrow) (**d**). Ten days later, retinal edema was reduced in both eyes (**f** and **h**) with obvious highly reflective exudates (arrow) in the macula of the right eye (**f**)

A 42-year-old woman presented with notable decreased vision and distortion in the right eye forty-two days after severe thoracoabdominal injury in a motorcycle accident. Visual acuity was 20/50 in the right eye and 20/63 in the left eye without improvement using a pinhole occluder. Anterior segment examinations were unremarkable. Fundus examination revealed dilated retinal veins, superficial hemorrhages, and multiple white retinal patches at the posterior pole in both eyes (Fig. 1a and b). The white retinal patches involved the macula of the right eye. FFA showed hyperpermeable vasculature and extensive fluorescence leakage in the middle and late stages (Fig. 2). OCT revealed highly reflective exudates in the neuroepithelium and macular edema in the right eye (Fig. 3). The patient had a history of strabismus surgery 16 years ago, and visual acuity had remained 20/63 in the left eye since then. A detailed history revealed that after the trauma, she underwent liver and bile duct surgery. Postoperatively, the abdominal incision was infected. Hemoculture revealed *Staphylococcus aureus* positivity, and intravenous antibiotics were started immediately. At the time of her first visit to the eye clinic, she was undergoing intravenous antibiotic therapy. Upon first glance at her fundal appearances with multiple white retinal patches and retinal hemorrhage, we considered it to be bilateral Purtscher’s retinopathy. No specific treatment was given to her.

Ten days later, the four separated retinal lesions in the right eye joined together with star-shaped hard exudates and radial folds in the macula (Fig. 1c). Visual acuity of the right eye decreased to 20/200. In the left eye, the color of the white retinal patches seemed lighter, and two cotton-wool spots near the optic disc disappeared (Fig. 1d). OCT revealed that retinal edema was reduced in both eyes (Fig. 3f and h) with obvious highly reflective exudates in the macula of the right eye. The decreased vision of the right eye and the change of the fundal appearances ten days later challenged our initial diagnosis of Purtscher’s retinopathy.

**Fig. 4:**
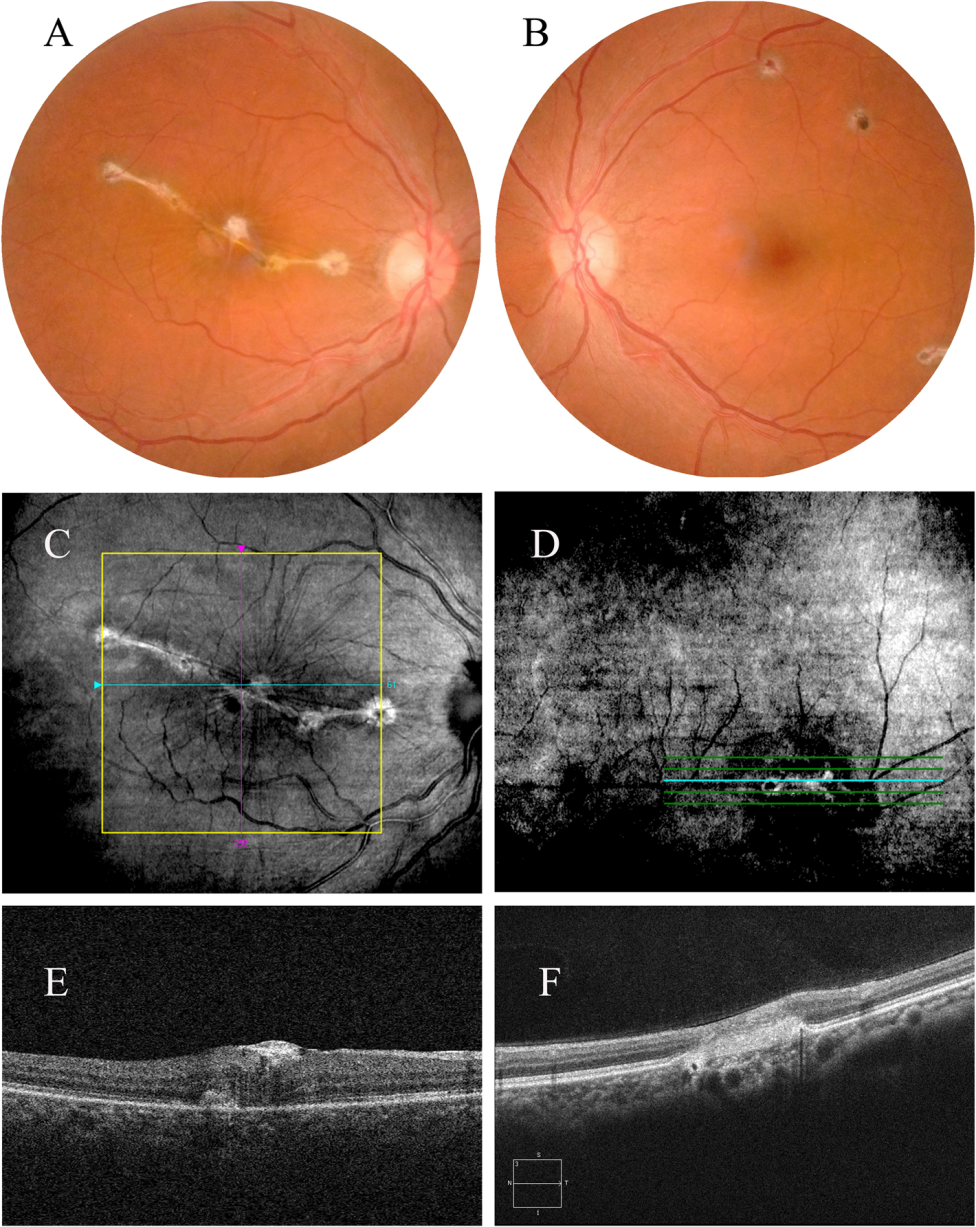
Fundus photographs (**a** and **b**) and optical coherence tomography (OCT) (**c**, **d**, **e** and **f**) of both eyes 2 years later. The photographs revealed cicatrization of the oval white patches in both eyes (**a** and **b**). In the right eye, macular scarring caused obvious radial folds (**a**). OCT showed fibrous tissue proliferation in the inner limiting membrane layer of the right eye (**c** & **e**). In the left eye, obvious subretinal fibrous tissue proliferation was found (**d** and **f**)

The patient was not followed up with until 2 years later. The BCVA of the right eye was 20/100. From her fundus photo (Fig. 4a and b), we found cicatrization of the oval white patches in both eyes. Especially in the right eye, macular scarring caused obvious radial folds that might have severely impaired visual function. OCT showed fibrous tissue proliferation in the inner limiting membrane layer of the right eye (Fig. 4c and e). In the left eye, obvious subretinal fibrous tissue proliferation was found (Fig. 4. d and f).

## Discussion and conclusions

Purtscher’s retinopathy is characterized by multiple superficial cotton-wool spots and flame-shaped retinal hemorrhages at the posterior pole in patients after severe compression injury to the head or trunk. It is named for the Austrian ophthalmologist, Othmar Purtscher (1852–1927), who detected it in 1910 and fully described it in 1912 [1]. Since its original description, Purtscher’s retinopathy has been associated with traumatic injury, primarily blunt thoracic and head trauma. When features similar to those observed with Purtscher’s retinopathy present without a history of trauma, it is called Purtscher-like retinopathy [5]. Purtscher-like retinopathy has been reported to occur in acute pancreatitis, renal failure, childbirth, retrobulbar anesthesia, and major cerebro- or cardiovascular surgery [6].

At first glance, we thought of the oval white retinal patches in this patient with thoracoabdominal trauma to be the cotton-wool spots of Purtscher’s retinopathy. First, in Purtscher’s retinopathy, cotton-wool spots usually appear 2–3 days after the trauma and fade away in four to 6 weeks. Decreases in vision are experienced at the same time. In this case, the patient complained of decreased vision forty-two days after the trauma. The onset time of vision decline and the white retinal patches were not consistent with that of Purtscher’s retinopathy. Second, cotton-wool spots are actually swollen nerve fibers caused by arteriolar nonperfusion, which can be confirmed by FFA. The cotton-wool spots should have the same shape and location as the nonperfusion area. In the present case, the four white retinal patches in the right eye were oval and located at the vascular ending in the posterior pole. In the left eye, there were also three oval white retinal patches. In addition, we saw three irregular white retinal lesions near the optic nerve head that were actually nonperfusion areas as demonstrated by FFA in the left eye. These three white lesions were cotton-wool spots. Third, in this case, FFA showed hyperpermeable vasculature instead of arteriolar nonperfusion in the oval white retinal lesions, and OCT revealed these yellowish lesions to be highly reflective exudates in the neuroepithelium and macular edema in both eyes. Therefore, the onset time, morphology, and location of the oval white retinal lesions demonstrated that this was not a simple case of bilateral Purtscher’s retinopathy.

Further history acquisition revealed that she suffered from *Staphylococcus aureus* bacteremia shortly before her first visit to the eye clinic and was undergoing intravenous antibiotic therapy. A case reported by Prajapati R demonstrated a striking subretinal abscess involving the macula in the right eye and an oval white-yellow retinal lesion in the left eye of an immunocompromised patient with *Staphylococcus aureus* septicaemia [7]. The retinal lesion in the left eye was consistent with the oval white retinal patches in our case. They were actually inflammatory exudates of focal endogenous endophthalmitis. As the patients received intravenous antibiotic therapy, the four separated retinal lesions joined together with star-shaped hard exudates and radial folds in the macula of the right eye. This was also consistent with the changes occurring in the subretinal abscess in the immunocompromised patient reported by Prajapati R. Therefore, this was actually a case of bilateral focal endogenous endophthalmitis.

However, the three irregular white patches at the posterior pole were not subretinal abscesses of endogenous endophthalmitis. FFA demonstrated them to be nonperfusion areas. As the patient had a history of trauma forty-two days before, we inferred that this might have been the resolution of Purtscher’s retinopathy. It might also be Purtscher’s-like retinopathy accompanied by endogenous endophthalmitis. Frequent causes of Purtscher’s-like retinopathy are acute pancreatitis, renal failure, and autoimmune disease [7]. However, Purtscher’s-like retinopathy has not been reported in infectious diseases.

For endogenous endophthalmitis, prolonged intravenous antibiotic treatment is necessary for the patient’s life and vision [8]. Therefore, the prompt and correct diagnosis of endogenous endophthalmitis is very important. At present, there is no consensus on the treatment of Purtscher’s and Purtscher’s-like retinopathy. Current treatments include glucocorticoid therapy, traditional Chinese medicine, and glucocorticoid integrative medicine therapy. A systematic review found that the difference between no treatment and glucocorticoid therapy showed no statistical significance [9]. In the present case, Purtscher’s or Purtscher’s-like retinopathy did not involve the macula of both eyes. The inflammatory exudates that developed secondary to systemic *Staphylococcus aureus* infection involved the macula and caused the decreased visual acuity and distortion in the right eye. As the patient was misdiagnosed with Purtscher’s retinopathy at her first visit, no specific treatment was given to her. Ten days later, we diagnosed her with focal endogenous endophthalmitis. She was given intravenous injections of ceftazidime, 4 g daily, for 10 days. Vitreous injection of antibiotics was not applied. Peripheral injection of triamcinolone acetonide was given to alleviate macular edema. The patient did not follow up as we had advised. However 2 years later, she returned with BCVA of the right eye at 20/100. Cicatrization of the oval white patches from her fundus photo and OCT image confirmed our diagnosis of endogenous endophthalmitis. Especially in the right eye, the macular scarring caused obvious radial folds that might have severely impaired visual function. We wondered whether the prognosis might have been better if vitreous injection of antibiotics and glucocorticoids had been applied.

Multiple retinal patches at the posterior pole and a history of trauma might easily lead to the diagnosis of Purtscher’s retinopathy. However, in the case of patients with multiple white retinal patches, especially those with infectious disease, we have to exclude the possibility of focal endogenous endophthalmitis. The correct diagnosis depends on reevaluation of the lesions by FFA and OCT, laboratory investigation and a detailed history.

## Data Availability

The datasets used and/or analysed during the current study are available from the corresponding author on reasonable request.

## Abbreviations

FFA: Fundus fluorescein angiography
OCT: Optical coherence tomography
